# Targeted Bisulfite Sequencing Reveals DNA Methylation Changes in Zinc Finger Family Genes Associated With KRAS Mutated Colorectal Cancer

**DOI:** 10.3389/fcell.2021.759813

**Published:** 2021-10-28

**Authors:** Weilin Pu, Fei Qian, Jing Liu, Keke Shao, Feng Xiao, Qin Jin, Qingmei Liu, Shuai Jiang, Rui Zhang, Jun Zhang, Shicheng Guo, Jianfeng Zhang, Yanyun Ma, Shaoqing Ju, Weifeng Ding

**Affiliations:** ^1^Department of Laboratory Medicine, Affiliated Hospital of Nantong University, Nantong, China; ^2^State Key Laboratory of Genetic Engineering, Collaborative Innovation Center for Genetics and Development, School of Life Sciences, Fudan University, Shanghai, China; ^3^Human Phenome Institute, Fudan University, Shanghai, China; ^4^Department of Gastrointestinal Surgery, Affiliated Hospital of Nantong University, Nantong, China; ^5^Department of Laboratory Medicine, The First People’s Hospital of Yancheng City, Yancheng, China; ^6^Department of Pathology, The Third People’s Hospital of Nantong City, Nantong, China; ^7^Department of Pathology, Affiliated Hospital of Nantong University, Nantong, China; ^8^Department of Dermatology, Huashan Hospital, Fudan University, Shanghai, China; ^9^Department of Gastroenterology, Huashan Hospital, Fudan University, Shanghai, China; ^10^Center for Precision Medicine Research, Marshfield Clinic Research Institute, Marshfield, WI, United States; ^11^Department of Gastroenterology, Affiliated Hospital of Nantong University, Nantong, China; ^12^Six Industrial Research Institute, Fudan University, Shanghai, China

**Keywords:** colorectal cancer, DNA methylation, zinc finger family, KRAS, diagnosis

## Abstract

**Background:** Colorectal cancer (CRC) is a leading cause of cancer death, and early diagnosis of CRC could significantly reduce its mortality rate. Previous studies suggest that the DNA methylation status of zinc finger genes (ZFGs) could be of potential in CRC early diagnosis. However, the comprehensive evaluation of ZFGs in CRC is still lacking.

**Methods:** We first collected 1,426 public samples on genome-wide DNA methylation, including 1,104 cases of CRC tumors, 54 adenomas, and 268 para-tumors. Next, the most differentially methylated ZFGs were identified and validated in two replication cohorts comprising 218 CRC patients. Finally, we compared the prediction capabilities between the ZFGs and the SEPT9 in all CRC patients and the KRAS + and KRAS- subgroup.

**Results:** Five candidate ZFGs were selected: *ESR1*, *ZNF132*, *ZNF229*, *ZNF542*, and *ZNF677*. In particular, *ESR1* [area under the curve (AUC) = 0.91] and *ZNF132* (AUC = 0.93) showed equivalent or better diagnostic capability for CRC than *SEPT9* (AUC = 0.91) in the validation dataset, suggesting that these two ZFGs might be of potential for CRC diagnosis in the future. Furthermore, we performed subgroup analysis and found a significantly higher diagnostic capability in KRAS + (AUC ranged from 0.97 to 1) than that in KRAS- patients (AUC ranged from 0.74 to 0.86) for all these five ZFGs, suggesting that these ZFGs could be ideal diagnostic markers for KRAS mutated CRC patients.

**Conclusion:** The methylation profiles of the candidate ZFGs could be potential biomarkers for the early diagnosis of CRC, especially for patients carrying KRAS mutations.

## Introduction

Colorectal cancer (CRC) is the second most common cause of cancer in the United States and accounts for 10% of cancer incidences and 9.4% of cancer deaths worldwide in 2020 (Siegel et al., 2020; Sung et al., 2021). Previous studies have found that the accumulations of both genetic and epigenetic alterations lead to CRC carcinogenesis. The 5-year survival rate of CRC ranges from 90% in its early stages (when it is localized and regional), but it decreases significantly to about 14% when detected at a distant stage, highlighting the importance of early detection methods (Siegel et al., 2020).

Recently, the application of screening modalities, including colonoscopy and image-based detection, significantly decreased the mortality of CRC (Siegel et al., 2017). However, these screening methods are not widely used across populations due to abdominal pain, discomfort, and other contraindications. Thus, the development of non-invasive and precise diagnostic methods for CRC is needed.

DNA methylation is a crucial epigenetic modification in the human genome and plays vital roles in embryonic development, transcription regulation (He et al., 2011; Jiang et al., 2018), and genomic imprinting (Schubeler, 2015). Moreover, the dynamic changes of DNA methylation in different tissues and disease courses make it a promising tool to develop the tissue-of-origin test (Guo et al., 2017a) and disease prediction (Guo et al., 2015), especially for cancers (Koch et al., 2018) and immune diseases (Guo et al., 2017b; Ding et al., 2018). Till now, A series of DNA methylation-based biomarkers have been found in CRC, including *SEPT9* (Barault et al., 2018; Freitas et al., 2018; Wills et al., 2018; Sun et al., 2019). However, the performance of *SEPT9* is not as good as that of the stool DNA test (Ahlquist et al., 2012; Church et al., 2014; Song et al., 2017). Therefore, identifying better DNA-methylation-based biomarkers with high accuracy would be beneficial in liquid biopsy in CRC.

Zinc finger proteins (ZFPs) are a prominent component of transcriptional factors in eukaryotes. The ZFP family can be divided into eight categories according to their distinct motifs: Cys2His2 (C2H2)-like, Gag knuckle, Treble clef, Zinc ribbon, Zn2/Cys6, TAZ2 domain-like, zinc-binding loops, and metallothionein (Jen and Wang, 2016). The C2H2-type zinc finger motifs form the largest class. Currently, growing bodies of evidence suggest that ZFPs could contribute to tumor progression or suppress it *via* transcriptional regulation. The DNA methylation alterations of multiple ZFPs have been recognized as promising biomarkers for tumor diagnosis, prognosis, and drug response due to their vital roles in cancers. However, a comprehensive and systemic assessment of the DNA methylation profiles of zinc finger genes (ZFGs) in CRC is lacking.

In this study, we exhaustively searched and combined public microarray datasets on high-throughput DNA methylation for the first time, including 1,104 CRC tumors, 54 adenomas, and 268 para-tumors. We identified seven candidate zinc finger genes using comprehensive filtering procedures, and five of them were successfully validated in 104 CRC Han Chinese patients, especially in the CRC tumors carrying the KRAS mutation. To confirm the findings, we recruited another 114 CRC patients of Han Chinese descent and yielded consistent results. In particular, two of these ZFGs, *ESR1*, and *ZNF132*, showed a significantly higher diagnostic capability than *SEPT9*, suggesting that they might be promising biomarkers for CRC diagnosis, especially for KRAS mutated patients.

## Results

### Five Zinc Finger Genes Were Identified as Candidate Colorectal Cancer Diagnostic Biomarkers

**FIGURE 1:**
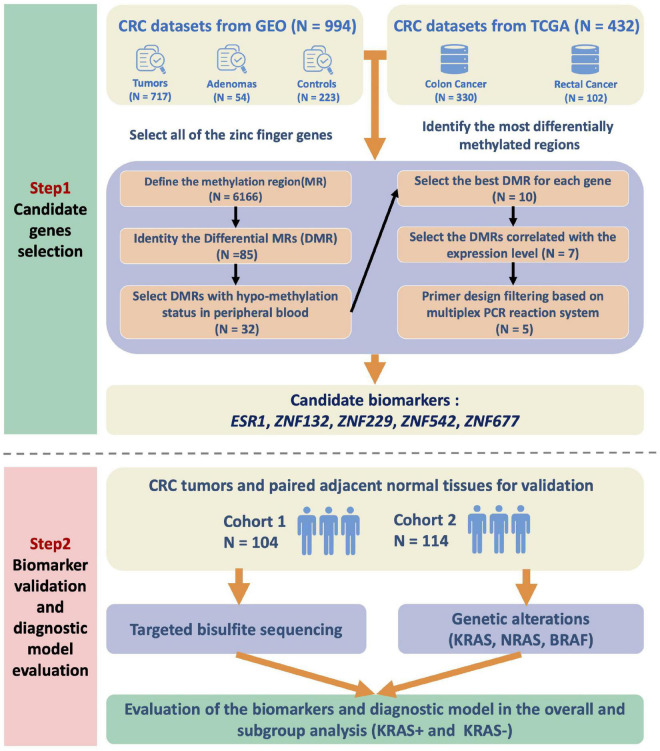
Flow chart of the study design. We first integrated the high-throughput DNA methylation microarray datasets from GEO and TCGA databases and examined the methylation profiles of all the zinc finger genes. To identify the most significantly differentially methylated genes in CRC, we first defined the methylation region and selected the differentially methylated regions (DMRs). The methylation status of the candidate DMRs in the whole blood, peripheral blood mononuclear cells and peripheral blood leukocytes were also utilized for biomarker filtering. The correlations between the methylation and expression level of the candidates were also calculated for further filtering. Finally, the candidate zinc finger genes-based biomarkers were validated in two independent CRC cohorts of the Han Chinese population. In addition, the KRAS mutation status of the patients was also detected and assessed for subgroup analysis.

To identify robust DNA methylation-based biomarkers, we searched the public datasets for the DNA methylation status in CRC cases and collected 1,104 CRC tumors, 268 para-tumor samples, and 54 adenomas for further analysis (See section “Materials and Methods,” Supplementary Table 1). We also obtained the complete list of genes belonging to the ZFP family (Supplementary Table 2). Based on the feature selection procedures described in the section “Materials and Methods,” we finally identified five candidate genes: *ESR1*, *ZNF132*, *ZNF229*, *ZNF542*, and *ZNF677* (Figure 1).

**FIGURE 2:**
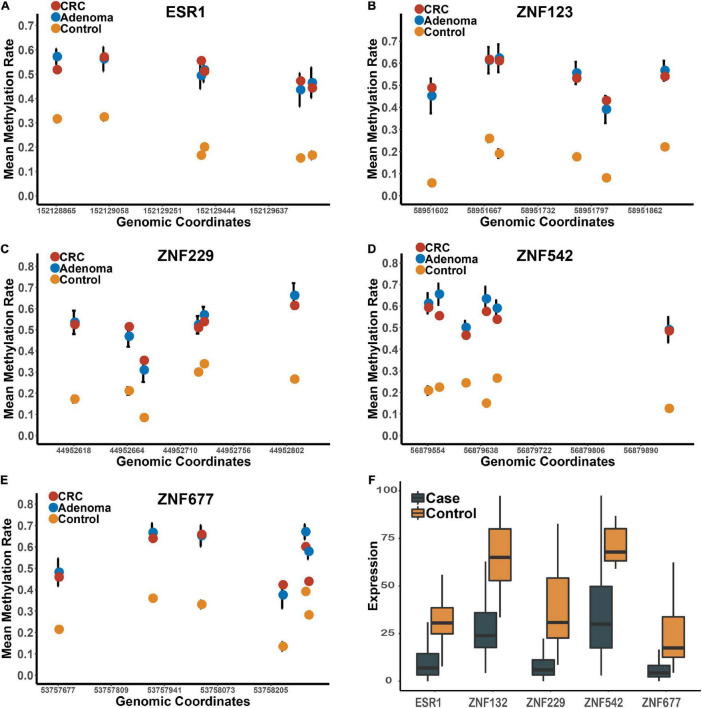
The methylation and expression profiles of the candidate biomarkers in the discovery datasets. Panels **(A–E)** represents the methylation profiles of the five candidates in the tumors, adenomas and para-tumors in the integrated dataset. Panel **(F)** represents the expression profiles of these five candidate zinc finger genes between CRC tumor and para-tumors in TCGA.

These five genes were significantly hypermethylated in both CRC and adenoma tissues compared to the para-tumors (Supplementary Figure 1). Consistently, the expression levels of these genes were also significantly down-regulated in CRC tumors compared to para-tumors in the TCGA dataset (Figure 2). To characterize the abilities of these biomarkers quantitatively in the combined discovery dataset, we constructed a univariate logistic regression model for each gene and obtained robust discrimination between CRC tumors and para-tumor tissues (sensitivity = 0.82–0.90, specificity = 0.88–0.97, AUC = 0.93–0.97).

### Targeted Bisulfite Sequencing in the Han Chinese Population Confirmed the Efficacy of the Candidate Zinc Finger Genes-Based Biomarkers

To further verify these DNA methylation-based biomarkers, we recruited 104 CRC patients of the Han Chinese population. The characteristics of these CRC patients (replication cohort 1) are shown in Table 1. The CRC tumors and their matched para-tumors were obtained for targeted bisulfite sequencing. The methylation profiles of the candidates and the known biomarker (*SEPT9*) were examined using targeted bisulfite sequencing (See section “Materials and Methods”). The bisulfite conversion rate (C to T) was high (>99%) in both tumors and para-tumors, and no significant difference in the read mapping rate was found between groups (Supplementary Figure 2). After quality control, 187 samples were retained for further analysis, including 98 CRC tumors and 89 para-tumors.

**TABLE 1:** Characteristics of the CRC patients included in this study.

**Characteristics**	**Patient distribution**	**Patient distribution**
	**Cohort 1 (*N* = 104)**	**Cohort 2 (*N* = 114)**
Age	66 (IQR = 62 to 74)	68 (IQR = 60 to 75)
**Gender**		
Male	71	75
Female	33	39
**Subtype^a^**		
Colon	55	68
Rectum	49	45
**UICC Stage^b^**		
I	18	28
II	35	38
III	40	38
IV	11	9
**Tumor invasion depth^c^**		
T1	5	7
T2	21	22
T3	70	69
T4	6	15
Tx	2	1
**Lymph node involvement^c^**		
N0	57	72
N1	29	25
N2	11	15
N3	5	2
Nx	2	
**Distant metastasis^c^**		
M0	93	105
M1	11	9
**KRAS mutation^d^**		
Positive	50	52
Negative	52	62

*^a^The tumors were classified into colon or rectum based on the location of the tumor.*

*^b^The UICC stage was determined after surgical intervention and histological examination of the specimen.*

*^c^TNM Stages were assessed by the seventh edition of the TNM classification criteria.*

*^d^The KRAS mutation status was examined using the FastTarget next-generation sequencing method.*

**FIGURE 3:**
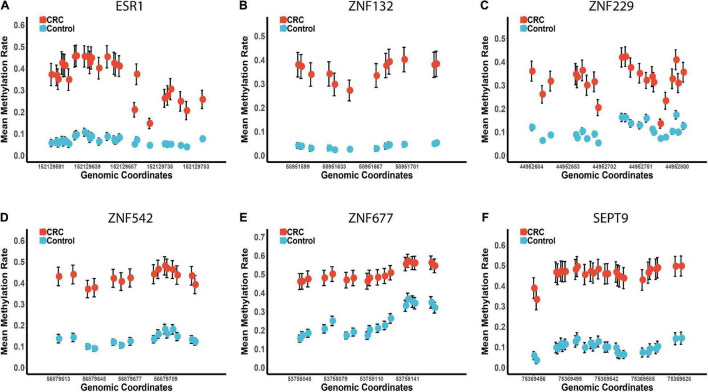
The methylation profiles of the five zinc finger genes and SEPT9 in the replication cohort 1. Panels **(A–F)** represents the mean methylation status of the CpG sites in regions covering *ESR1*, *ZNF132*, *ZNF229*, *ZNF542*, *ZNF677*, and *SEPT9*, respectively. The x-axis represents the genomic positions of the CpG sites in the targeted regions. The y-axis represents the mean methylation percentage in the CRC tumor tissues and paired normal tissues for each CpG site. The error bar of each CpG site represents the confidence interval of the methylation percentage.

Based on the methylation profiles of these five ZFGs, we performed principal component analysis (PCA) and found a significant distinction between CRC tumors and para-tumors (Supplementary Figure 3). A differential methylation analysis was also conducted for these candidates, and we found that all candidate genes were significantly hypermethylated in CRC tumors (Figure 3 and Supplementary Figure 4). Furthermore, we performed a univariate logistic regression analysis without adjusting for covariates, and created the receiver operating characteristic (ROC) curve to reveal the diagnostic ability of each candidate gene. As shown in Supplementary Table 3, the area under the curve (AUCs) of these candidates ranged from 0.85 to 0.93. In particular, we found that the diagnostic capability of *ZNF132* (AUC = 0.91) and *ESR1* (AUC = 0.93) was equal to or better than that of *SEPT9* (AUC = 0.91), indicating that these ZFGs might have great potential for CRC diagnosis.

### The Diagnostic Abilities of the Candidates Were Significantly Affected by the KRAS Mutation Status of Colorectal Cancer Patients

**FIGURE 4:**
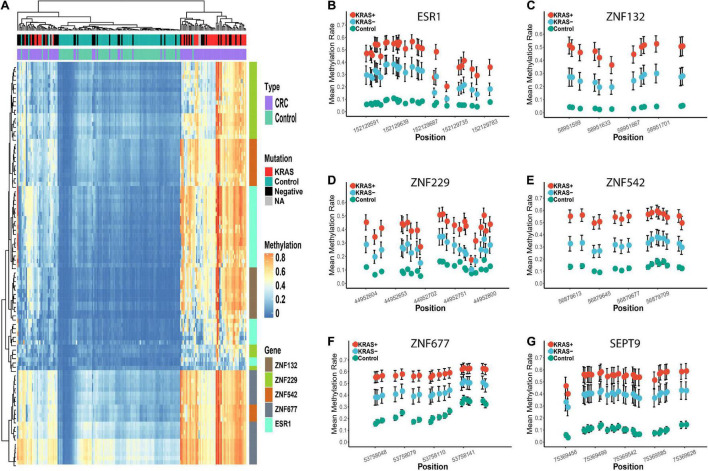
Differential methylation profiles of these candidate biomarkers in KRAS + and KRAS- subgroup in replication cohort 1. Panel **(A)** represents the heatmap of the methylation levels of these five candidate genes. The tumor and para-tumors tissues were differently colored. The KRAS mutation status of the CRC tumors is also differently colored. Panels **(B–G)** represents the methylation profiles of the five zinc finger genes and SEPT9. The mean methylation percentage of each CpG site is shown for KRAS +, KRAS- and control groups.

In addition to the overall differential methylation analysis, we also evaluated the effects of age, gender, tumor stage, location of the tumor (colon or rectum), and the mutation status (KRAS + vs. KRAS-) of the CRC samples (Supplementary Tables 4–8). No significant differences were found in the diagnostic capability between CRC patients in the young/old, male/female, early/late stage, and colon/rectum subgroups (Supplementary Table 9, see section “Materials and Methods”). However, the diagnostic capability for CRC patients carrying the KRAS mutation (KRAS +) was significantly better than that for KRAS- samples (Supplementary Tables 8, 9). In the KRAS + group, the sensitivity of each gene ranged from 0.86 to 0.98, the specificity range was 0.89 to 1.00, and the AUC range was 0.97 to 1.00, which is significantly higher than the sensitivity (0.52 to 0.82), specificity (0.80 to 0.98) and AUC (0.74 to 0.86) in the KRAS- group (Supplementary Table 8). Moreover, the hierarchical clustering analysis revealed that all CRC tumors misclassified into the control group were KRAS- samples (Figure 4). In addition, we further examined if other factors may be associated with these misclassified CRC tumors (*n* = 17). However, none of the age, gender, tumor stage, and tumor locations showed significant differences in our study’s overall CRC tumors. Furthermore, we examined the diagnostic abilities of the ZFGs in the KRAS + and KRAS- subgroup from the TCGA dataset for verification. Consistently, the diagnostic capability of the ZFGs in the KRAS + subgroup (AUC = 0.96–1) was significantly higher than in the KRAS- subgroup (AUC = 0.93–0.97), suggesting the significant methylation changes between KRAS + and KRAS- subgroup (Supplementary Table 10).

### An Independent Replication Cohort Validated the Association Between KRAS Mutation and Diagnostic Capabilities of the Zinc Finger Genes

Due to the limited sample size in replication cohort 1, we recruited another sample group (replication cohort 2), including 114 CRC tumors and matched para-tumors from patients of Han Chinese descent (Table 1) for further validation. The methylation profiles of these ZFGs were measured in replication cohort 2 (Supplementary Figures 5, 6). Consistently, we found that *ESR1* (AUC = 0.90) and *ZNF132* (AUC = 0.94) still showed the best diagnostic capability for CRC (Supplementary Table 11).

**FIGURE 5:**
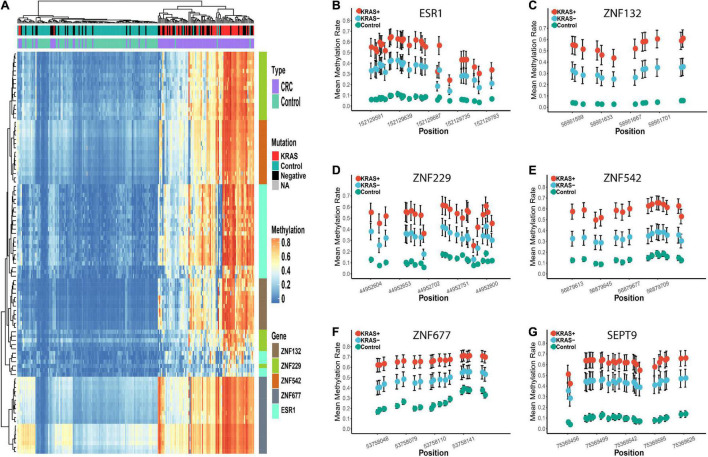
Differential methylation profiles of these candidate biomarkers in KRAS + and KRAS- subgroup in replication cohort 2. Panel **(A)** represents the heatmap of the methylation levels of these five candidate genes. The tumor and para-tumors were differently colored. The KRAS mutation status of the CRC tumors is also differently colored. Panels **(B–G)** represents the methylation profiles of the five zinc finger genes and SEPT9. The mean methylation percentage of each CpG site is shown for KRAS +, KRAS- and control groups.

Furthermore, the significant differences of methylation profiles between the KRAS + and KRAS- subgroups were also validated (Supplementary Figure 7). In the KRAS + subgroup, the sensitivity (0.90 to 1.00), specificity (0.91 to 0.98), and the AUC (0.92 to 1.00) of each gene was significantly higher than that in the KRAS- subgroup (sensitivity: 0.58 to 0.85, specificity: 0.78 to 0.96, AUC: 0.71 to 0.88) (Supplementary Table 12). Similarly, the CRC tumors misclassified as the para-tumors were in the KRAS- subgroup, confirming that the KRAS + CRC samples were more epigenetically homogeneous than the KRAS- CRC samples (Figure 5).

### The Diagnostic Abilities of Zinc Finger Genes Could Achieve Superior Performance Than That of *SEPT9* for Colorectal Cancer Diagnosis Using All Replication Samples

**FIGURE 6:**
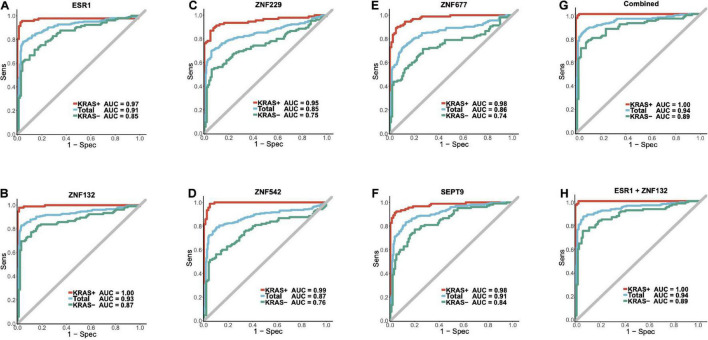
The diagnostic abilities for the candidate biomarkers and ZFGx model in the combined replication cohort. Panels **(A–F)** represents the diagnostic ability of *ESR1*, *ZNF132*, *ZNF229*, *ZNF542*, *ZNF677*, *SEPT9* individually. Panel **(G)** represents the ROC curve of the diagnostic model using all five candidate zinc finger genes (*ESR1*, *ZNF132*, *ZNF229*, *ZNF542*, *ZNF677*). Panel **(H)** represents the ROC curve of the ZFGx model using only *ESR1* and *ZNF132* as variables. The diagnostic ability of each model for the overall samples, KRAS + and KRAS- subgroups are shown.

To further identify the robust diagnostic biomarker for CRC diagnosis, we combined the datasets from replication cohort 1 and replication cohort 2. As shown in Figure 6, *ZNF132* had the highest diagnostic capability (sensitivity = 0.83, specificity = 0.97, AUC = 0.93) than other genes, including *SEPT9* (sensitivity = 0.83, specificity = 0.87, AUC = 0.91). In addition, *ESR1* also achieved comparable diagnostic capability (sensitivity = 0.78, specificity = 0.97, AUC = 0.91) to *SETP9* (Table 2), suggesting that these two genes have great potential in the liquid biopsy of CRC. Simultaneously, we assessed the diagnostic abilities of these ZFGs in the KRAS + subgroups and KRAS- group using all samples. Concordantly, all candidate biomarkers achieved better performance (AUC ≥ 0.95) in the KRAS + group than that in the KRAS- group, further indicating that KRAS- CRC patients are more epigenetically homogeneous.

**TABLE 2:** The mean methylation status of the five genomic regions in the KRAS + and KRAS- samples of replication cohort 1 and cohort 2.

	**Genomic Region^a^**	**Gene^b^**	**McaM^c^**	**McoM^c^**	***P* value^d^**	**FDR**	**OR**	**95% CI^e^**	**Sens^f^**	**Spec^f^**	**AUC^f^**
KRAS +	6:152129591-152129791	*ESR1*	0.49	0.06	8.00 × 10^–27^	9.60 × 10^–27^	11.00	7.53–15.60	0.96	0.98	0.97
	19:58951599-58951728	*ZNF132*	0.51	0.03	2.10 × 10^–29^	1.20 × 10^–28^	29.00	17.6–47.90	0.98	0.99	1.00
	19:44952604-44952808	*ZNF229*	0.47	0.11	1.70 × 10^–24^	1.70 × 10^–24^	10.20	7.19–14.00	0.90	0.93	0.95
	19:56879613-56879735	*ZNF542*	0.57	0.13	4.00 × 10^–29^	1.20 × 10^–28^	14.30	9.60–21.50	0.99	0.95	0.99
	19:53758048-53758164	*ZNF677*	0.63	0.25	3.40 × 10^–27^	5.10 × 10^–27^	10.90	7.64–15.30	0.90	0.95	0.98
	17:75369456-75369630	*SEPT-9*	0.58	0.08	2.40 × 10^–27^	4.70 × 10^–27^	7.85	5.55–11.00	0.91	0.96	0.98
KRAS-	6:152129591-152129791	*ESR1*	0.31	0.08	2.60 × 10^–18^	7.90 × 10^–18^	4.63	3.21–6.39	0.75	0.83	0.85
	19:58951599-58951728	*ZNF132*	0.28	0.04	1.30 × 10^–19^	7.70 × 10^–19^	6.10	4.09–8.73	0.70	0.97	0.87
	19:44952604-44952808	*ZNF229*	0.29	0.12	6.90 × 10^–10^	8.20 × 10^–10^	3.30	2.20–4.60	0.54	0.94	0.75
	19:56879613-56879735	*ZNF542*	0.33	0.15	1.50 × 10^–10^	2.30 × 10^–10^	2.78	1.87–3.84	0.50	0.96	0.76
	19:53758048-53758164	*ZNF677*	0.46	0.27	1.80 × 10^–09^	1.80 × 10^–09^	2.38	1.62–3.25	0.71	0.73	0.74
	17:75369456-75369630	*SEPT-9*	0.41	0.11	2.30 × 10^–17^	4.70 × 10^–17^	2.94	2.11–3.92	0.77	0.80	0.84
Total	6:152129591-152129791	*ESR1*	0.39	0.07	1.20 × 10^–45^	3.50 × 10^–45^	6.06	4.81–7.52	0.78	0.95	0.91
	19:58951599-58951728	*ZNF132*	0.39	0.04	6.30 × 10^–49^	3.80 × 10^–48^	8.67	6.60–11.20	0.83	0.97	0.93
	19:44952604-44952808	*ZNF229*	0.37	0.11	3.50 × 10^–33^	3.50 × 10^–33^	5.04	3.99–6.24	0.70	0.94	0.85
	19:56879613-56879735	*ZNF542*	0.44	0.14	1.00 × 10^–37^	1.50 × 10^–37^	4.42	3.56–5.39	0.79	0.90	0.87
	19:53758048-53758164	*ZNF677*	0.54	0.26	1.60 × 10^–34^	1.90 × 10^–34^	3.85	3.11–4.67	0.73	0.90	0.86
	17:75369456-75369630	*SEPT-9*	0.49	0.10	5.50 × 10^–45^	1.10 × 10^–45^	4.02	3.27–4.02	0.83	0.87	0.91

*^a^Genomic region represents the genomic coverage of the reads with targeted bisulfite sequencing, and the genomic coordinates shown here is based on the hg19 version of the genome.*

*^b^The gene name of the genomic region.*

*^c^McaM represents the mean methylation percentage of the cases in each region, which consisting of several CpG sites, while the McoM represents the mean methylation percentage of the controls in each region.*

*^d^P value is calculated through the wilcoxon rank-sum test following with FDR (false discovery rate) adjustment for multiple corrections.*

*^e^OR and 95% CI were conducted through logistic regression.*

*^e^Sens, sensitivity; while Spec, specificity; AUC, area under curve.*

*^f^The sensitivity, specificity, and the AUC were calculated through a logistic regression prediction model without adjustment for gender, age and smoking status and alcohol status.*

To further improve the robustness of the diagnostic abilities of these ZFGs, we performed multiple machine-learning algorithms following the fivefold cross-validation method, which split the dataset into training and validation dataset randomly to obtain an unbiased estimation (see section “Materials and Methods,” Supplementary Table 13). As shown in Supplementary Table 13, the random forest (RF) model achieved the best accuracy (accuracy = 0.89) in the test dataset. In the KRAS + subgroup, the Naïve Bayes algorithm performed best on the test dataset (sensitivity = 0.99, specificity = 0.97, accuracy = 0.98), while the Neural Network model had the best performance in the KRAS- subgroup (sensitivity = 0.75, specificity = 0.89, accuracy = 0.82). Therefore, we suggested that the diagnostic abilities of these ZFGs are reliable for CRC diagnosis in test dataset.

## Discussion

To our knowledge, few studies have extensively explored the methylation alterations of ZFGs in CRC. Herein, we integrated datasets from TCGA and GEO databases to identify robust and statistically powerful biomarkers. In total, we identified five hyper-methylated ZFGs as candidate biomarkers and validated them using Han Chinese CRC patients. Two ZFGs, *ZNF132*, and *ESR1*, were recognized as promising diagnostic biomarkers for CRC. Moreover, we found significantly higher diagnostic abilities of these ZFGs in the KRAS + group than the KRAS- group, suggesting the significant association between somatic mutations and DNA methylation alterations. Our results highlighted the importance of combining genetic mutations and epigenetic alterations for CRC diagnosis in further studies.

The interaction between genetic mutations and epigenetic alterations in the tumorigenesis of CRC has been reported previously. Gazin et al. (2007) performed a genome-wide RNA interference (RNAi) screening of K-ras-transformed NIH 3T3 cells and identified 28 genes that are required for Ras-mediated epigenetic silencing of the pro-apoptotic Fas gene. It was suggested that Ras-mediated epigenetic silencing could lead to CRC oncogenesis through the epigenetic inactivation of key genes. Nagasaka et al. also found that both *KRAS* and *BRAF* mutation could contribute to the global hypermethylation phenotype of CIMP genes in colon cancer. Furthermore, Serra et al. (2014) revealed that *KRAS* mutation could result in the hypermethylation and transcriptional silencing of CIMP genes through *ZNF304*, indicating the importance of ZFPs in the carcinogenesis of CRC. In our study, the methylation profiles of the five ZFGs were significantly associated with the *KRAS* mutation status, suggesting that *KRAS* mutation may alter the downstream pathways through the epigenetic regulation of these ZFGs and required further verification.

Among the five ZFGs, *ZNF132* was identified as the most promising biomarker for CRC diagnosis in our analysis. *ZNF132* is located at 19q13.4 and belongs to the C2H2 ZFP family (Tommerup and Vissing, 1995). Previous studies have identified the DNA methylation alterations of *ZNF132* in breast cancer, esophageal squamous cell carcinoma (ESCC), oropharyngeal squamous cell carcinoma, and prostate cancer (Lleras et al., 2011; Abildgaard et al., 2012; Stefansson et al., 2015; Jiang et al., 2018). It is reported that *ZNF132* hypermethylation could reduce the Sp1 transcript factor activity and decrease the growth, migration, invasion, and tumorigenicity capabilities of cells in a nude mouse model of ESCC (Jiang et al., 2018). Our study identified the hypermethylation and down-regulation of *ZNF132* in CRC, especially in KRAS-mutated samples, suggesting its biological implications in CRC tumorigenesis.

*ESR1* (estrogen receptor alpha 1) has been recognized as a tumor-suppressor gene and an estrogen receptor gene. It encodes the main mediator of estrogen effects in breast epithelial cells and has also been shown to be activated by epidermal growth factor (EGF). The hyper-methylation status of *ESR1* has been reported previously in lung adenocarcinoma, breast cancer, prostate cancer, squamous cell cervical cancer, and CRC (Li et al., 2004; Lin et al., 2009; Kim et al., 2010; Elliott et al., 2013; Kirn et al., 2014; Martinez-Galan et al., 2014). *ESR1* hypermethylation is also correlated with poor prognosis and drug response in breast cancer (Ramos et al., 2010; Mastoraki et al., 2018). Additionally, the *ESR1* promoter hypermethylation has been associated with KRAS mutation, which is consistent with our results (Horii et al., 2009).

*ZNF229* is a protein-coding gene, and few studies have suggested the hypermethylation status of *ZNF229* in cancer diagnosis. The biological functions and their role in CRC tumorigenesis should be explored further. *ZNF542* may involve in the epigenetic regulation of puberty through transcriptional repression (Lomniczi et al., 2015). Moreover, a CpG site located at *ZNF542* has been found to be a promising biomarker for ESCC (Pu et al., 2017). A pan-cancer study revealed that *ZNF542* was significantly hypermethylated in 10 kinds of cancers (Gevaert et al., 2015). *ZNF677* is located at chromosomal region 19q13 and was found to regulate the putative tumor cell growth suppressor in non-small cell lung cancers through hypermethylation (Heller et al., 2015). In addition, *ZNF677* is frequently down-regulated by promoter methylation in primary papillary thyroid cancers (PTC), and the decreased expression of *ZNF677* is significantly correlated with poor survival (Li et al., 2018).

Currently, most CRC patients are still diagnosed at later stages, especially in developing countries, and there is an urgent need for better diagnostic biomarkers. DNA methylation alterations may occur before mRNA and protein changes and could thus be ideal for early diagnosis. *SEPT9* has been approved as a DNA methylation-based diagnostic biomarker for CRC. The first release of the cfDNA *SEPT9* DNA methylation assay achieved considerable sensitivity (72%) and specificity (86%) in CRC detection using plasma (deVos et al., 2009). Since then, an updated version of the assay (Epi proColon 2.0) has shown better sensitivities (68–95%) and specificities (80–99%) in CRC diagnosis (Payne, 2010). However, the significant heterogeneity of CRC makes it challenging to use a single DNA methylation-based biomarker to diagnose CRC accurately. Therefore, the present study showed that *ZNF132* and *ESR1* have comparable or even better diagnostic capability than *SEPT9*, suggesting that the panel integrating *ZNF132, ESR1, and SEPT9* may better serve as a robust non-invasive diagnostic tool for CRC. In addition, the diagnostic ability of our model and the *SEPT9* model were both significantly affected by the KRAS mutation status in patients, and none of them achieved satisfactory sensitivities and specificities in the diagnosis of KRAS- CRC patients, suggesting that the mutation landscape of the patients should be taken into account to diagnose CRC accurately.

We acknowledge that our study has several limitations. First, our study’s sample size and patient diversity are limited, and studies with larger sample size and diverse populations are required to identify better CRC diagnostic markers. Second, we identified the candidate ZFGs by analyzing public DNA methylation datasets using Illumina HumanMethylation 450K microarray, which only covered a small proportion of the genome while a large amount of the genome remains undetected. Further studies using whole-genome bisulfite sequencing may be required to identify better DNA methylation-based biomarkers for CRC. Finally, our study only detected the methylation profiles of these ZFGs in tumor or para-tumor tissues, while the efficacy of these ZFGs for CRC non-invasive diagnosis using plasma or stool is elusive. We will try to validate the diagnostic abilities of these ZFGs in our future studies.

## Materials and Methods

### Integration of Public Datasets and Biomarker Discovery

Public high-throughput DNA methylation microarray datasets (Illumina HumanMethylation 450K) were searched exhaustively from TCGA and GEO databases. Two datasets from TCGA and nine datasets from GEO were included, yielding a total of 1,104 CRC tumor samples, 268 control samples, and 54 adenoma samples (Supplementary Table 1). The comprehensive list of genes in the zinc finger family (*n* = 1,594) was obtained from HGNC (Supplementary Table 2).

As shown in a previous study, the methylation linkage equilibrium decreased significantly when the block was longer than 1,000 bp (Guo et al., 2017a). Therefore, we defined the methylation region (MR) to have at least 6 CpG sites with less than 1,000 bp. We then arranged all the CpG sites in the high-throughput microarray according to their genomic coordinates and applied the sliding window method to identify all MRs. In total, we identified 6,166 MRs and performed differential methylation analyses (Supplementary Table 14). The 85 DMRs (McaM > 0.50, MadM > 0.50 and McoM < 0.30) were found for further analysis. To correct the noise from DNA originating from mixed-in peripheral blood, we suggested that the methylation rate of the DMRs need to be extremely low in the peripheral blood. Thus, we integrated the public high-throughput microarray datasets of the whole blood (WB, *n* = 1438), peripheral blood mononuclear cells (PBMC, *n* = 111), and peripheral blood leukocytes (PBL, *n* = 529) as references for DMR identification (Supplementary Table 15). The 32 DMRs were retained due to their low methylation rates in WB, PBMC, or PBL (mean methylation rate < 0.10).

Several DMRs were located at the same gene, and we selected the DMR with the most significant differences between CRC and control tissues for each gene (*N* = 10). To obtain the DMRs that may regulate the expression of neighboring genes, we further selected the DMRs with transcription factor binding sites (TFBSs) that correlate significantly with the expression of neighboring genes (Supplementary Tables 16, 17). In total, seven out of the ten candidate DMRs were selected for validation. However, due to the difficulties in the primer design due to CG percent, PolyT, and the number of SNPs, two candidate DMRs were removed (*SALL1* and *ZSCAN23*). Finally, we obtained the top five candidate DMRs for further validation (*ESR1*, *ZNF132*, *ZNF229*, *ZNF542*, and *ZNF677*).

### Patients, Samples, and DNA

Colorectal cancer patients in both replication cohort 1 and cohort 2 were recruited from the Affiliated Hospital of Nantong University between 2016 and 2018. The earlier recruited samples (*N* = 104) were included in replication cohort 1, while the others belonged to replication cohort 2. The recruited patients with available CRC tumors and matched para-tumors (the corresponding adjacent normal tissue at least 5 cm distant from the tumor tissue) were selected in this study. The patients recruited had not been treated with any neoadjuvant therapy before. Tumors were classified according to TNM (Tumor Node Metastasis)/UICC (Union for International Cancer Control) criteria following the histopathological examination. At least two professional pathologists evaluated all tumor samples carefully. All procedures performed in this study were in accordance with the ethical standards of the institutional research committee, as well as the 1964 Declaration of Helsinki and its later amendments. The study was approved by the institutional review boards of the Affiliated Hospital of Nantong University. Written informed consent was obtained from each participant of the study. All tumors and para-tumors were immediately frozen at −80°C after surgical resection.

### Targeted Bisulfite Sequencing Assay and Detection of *KRAS*, *NRAS*, and *BRAF* Mutation Status

DNA extraction was performed using the AIIperp DNA/RNA Mini Kit (Qiagen, Duesseldorf, Germany) according to the manufacturer’s protocols. The EpiTect Fast DNA Bisulfite Kit (Qiagen, Duesseldorf, Germany) was further used for bisulfite conversion. After carefully evaluating CG percent, PolyT, and the occurrence of SNPs in the targeted regions of the candidate DMRs, we designed primers to detect them in a panel for NGS sequencing (Supplementary Table 18). The PCR amplicons were then diluted and amplified using these primers, and the products (170–270 bp) were further separated and purified by QIAquick Gel Extraction kit (Qiagen, Duesseldorf, Germany). Finally, the sequencing libraries from different samples were pooled together and sequenced using the Illumina Hiseq 2000 platform according to the manufacturer’s protocols.

The BSseeker2 was applied for read mapping and methylation calling. After that, we removed the samples with a bisulfite conversion rate < 98%. The methylation level of each CpG site for each sample has been provided in Supplementary Tables 19, 20. The average coverage and missing rate for each CpG site were calculated and utilized for quality control (average coverage > 20X, missing rate < 20%). In addition, the sample whose missing rate > 30% were also filtered out. FastTarget next-generation sequencing was used to detect tumor DNA for the mutations in codons 12, 13, 59, 61, 117, and 146 of the *KRAS* and *NRAS* genes, as well as the mutation in codon 600 of the *BRAF* gene (Liao et al., 2019).

### Statistical Analysis and Machine Learning Methods

The Wilcoxon rank-sum test was performed in the discovery stage to identify the differential methylation sites and regions between CRC tumors, adenomas, and para-tumors. Moreover, the differential methylation status (odds ratios) between tumors and para-tumors of the DMRs were calculated with logistic regression. Benjamini-Hochburg correction was utilized for multiple test correction.

To identify the factors that may affect the diagnostic abilities of these ZFGs in CRC, we first split the dataset based on these covariates, including young (age ≤ median age) vs. old (age > median age), male vs. female, early (Stage I/II) vs. late (Stage III/IV), colon vs. rectum and KRAS + vs. KRAS- subgroups. Second, we performed the univariate logistic regression model using each gene in these subgroups separately. The cut-off value was determined as the value which maximized the sum of both sensitivity and specificity of the model. After that, the predicted sample type for each sample was obtained, and was used to calculate the number of accurate predictions (true positive + true negative) and inaccurate predictions (false positive + false negative) of the logistic model. Finally, we utilized the Fisher exact test to explore if there are significant differences between the prediction outcomes between these subgroups.

To further give an unbiased estimation of the diagnostic ability of these ZFGs, we performed fivefold cross-validation of our dataset using the logistic regression (Package stats), support vector machine (SVM, Package e1071), random forest (Package randomForest), Naïve Bayes (Package e1071), neural network (Package nnet), linear discriminant analysis (LDA, Package mda), mixture discriminant analysis (MDA, Package mda), flexible discriminant analysis (FDA, Package mda), gradient boosting machine (Package gbm), catboost (Package catboost), and XGBoost (Package xgboost) methods. We randomly selected 80% of our samples as the training dataset, and the remaining 20% of samples were used as the test dataset. After that, we optimized these machine learning methods using the training dataset and validate these optimized models in the test dataset. We repeated these procedures 1,000 times, and obtained the sensitivities, specificities and accuracies for both training and test datasets. The averaged sensitivities, specificities and accuracies for both training and test datasets were calculated and shown in Supplementary Table 13. All statistical analyses were conducted using R (v3.4.3).

## Data Availability Statement

Datasets related to this article can be found at Zenodo (doi: 10.5281/zenodo.5214274) and in Supplementary Tables 19, 20. The analysis steps, functions, and parameters used are described in detail in section “Materials and Methods”. The custom R scripts used to analyze data and generate figures are available at a GitHub repository: https://github.com/puweilin/CRC_MethylationAnalysis.git.

## Ethics Statement

The studies involving human participants were reviewed and approved by the institutional review boards of the Affiliated Hospital of Nantong University. The patients/participants provided their written informed consent to participate in this study.

## Author Contributions

WD, WP, SG, YM, and SJ contributed to the conception and design of the study. WD, FQ, KS, FX, QJ, SJ, RZ, JZ, JFZ, and SQJ collected the samples of the study. FQ, WD, and SQJ performed the experiments of this study. WP, WD, FQ, JL, YM, and SJ performed the statistical analysis. WP and WD wrote the first draft of the manuscript. SJ, YM, and SG revised the manuscript. All authors contributed to manuscript revision, read, and approved the submitted version.

## Conflict of Interest

The authors declare that the research was conducted in the absence of any commercial or financial relationships that could be construed as a potential conflict of interest.

## Publisher’s Note

All claims expressed in this article are solely those of the authors and do not necessarily represent those of their affiliated organizations, or those of the publisher, the editors and the reviewers. Any product that may be evaluated in this article, or claim that may be made by its manufacturer, is not guaranteed or endorsed by the publisher.

## Supplementary Material

Supplementary Figure 1 The Mean methylation rate of the candidate zinc finger genes in the CRC tumors, adenomas and control tissues. The y-axis represents the mean methylation percentage in the CRC tumor tissues and paired normal tissues for each CpG site. The error bar of each CpG site represents the confidence interval of the methylation percentage.

Supplementary Figure 2 Bisulfite conversion rate and mapping rate between CRC tumors and para-tumors. Panels **(A,B)** represent the bisulfite conversion rate and the reads mapping rate of the samples in replication cohort 1, respectively.

Supplementary Figure 3 Principal Component Analysis of the methylation profiles of the samples in replication cohort 1. All of the CpG sites were preprocessed and simplified into the two-variable space through principal component analysis. The green and red dots represented the CRC tumors and paired controls in replication cohort 1.

Supplementary Figure 4 Boxplot of the methylation rate of the KRAS +, KRAS- and control samples in replication cohort 1. The mean methylation rate of all the CpG sites in each candidate gene of each sample was depicted as one dot in the boxplot.

Supplementary Figure 5 Bisulfite conversion rate and mapping rate between CRC tumors and para-tumors. Panels **(A,B)** represent the bisulfite conversion rate and the reads mapping rate of the samples in replication cohort 2, respectively.

Supplementary Figure 6 Principal Component Analysis of the methylation profiles of the samples in replication cohort 1. All of the CpG sites were preprocessed and simplified into the two-variable space through principal component analysis. The green and red dots represented the CRC tumors and paired controls in replication cohort 2.

Supplementary Figure 7 Boxplot of the methylation rate of the KRAS +, KRAS- and control samples in replication cohort 2. The mean methylation rate of all the CpG sites in each candidate gene of each sample was depicted as one dot in the boxplot.
