# Supplementary figures and images for: Targeted Bisulfite Sequencing Reveals DNA Methylation Changes in Zinc Finger Family Genes Associated With KRAS Mutated Colorectal Cancer
Source: Front Cell Dev Biol. 2021 Oct 28;9:759813. doi: 10.3389/fcell.2021.759813 (PMC8581662; doi:10.3389/fcell.2021.759813)

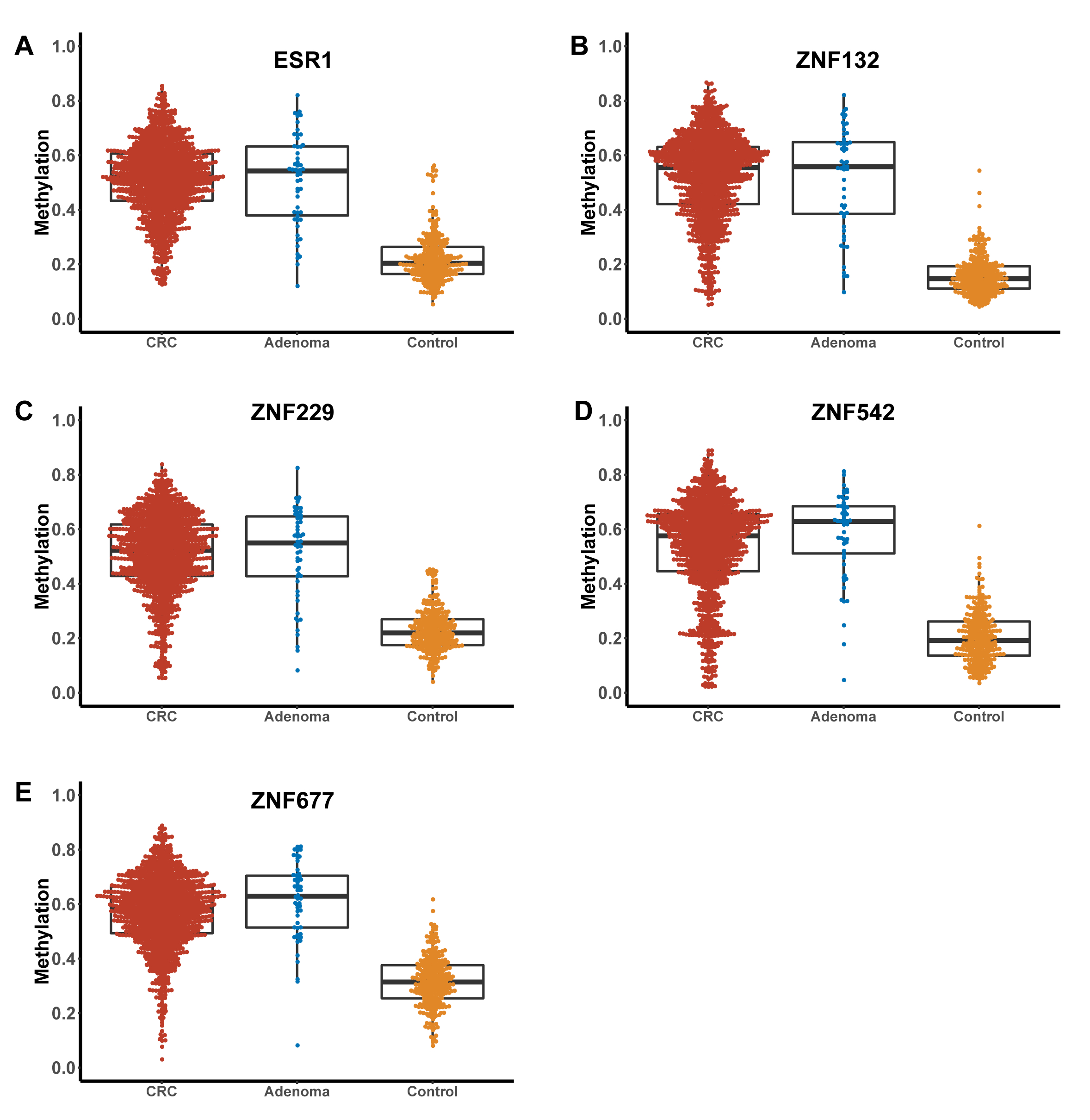

Supplement: Supplementary Figure 1 — The Mean methylation rate of the candidate zinc finger genes in the CRC tumors, adenomas and control tissues. The y-axis represents the mean methylation percentage in the CRC tumor tissues and paired normal tissues for each CpG site. The error bar of each CpG site represents the confidence interval of the methylation percentage. [file Image_1.JPEG]

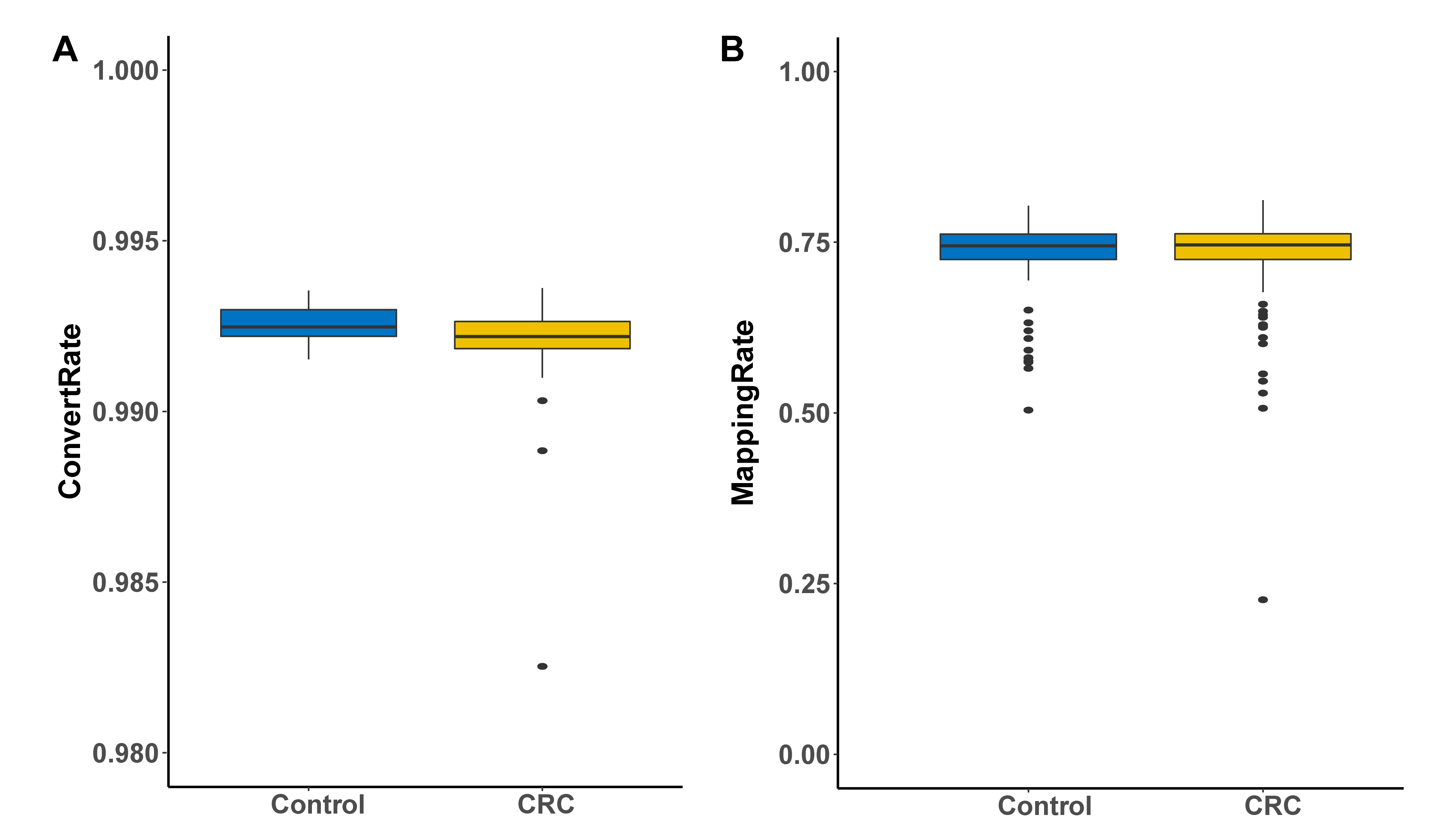

Supplement: Supplementary Figure 2 — Bisulfite conversion rate and mapping rate between CRC tumors and para-tumors. Panels (A,B) represent the bisulfite conversion rate and the reads mapping rate of the samples in replication cohort 1, respectively. [file Image_2.JPEG]

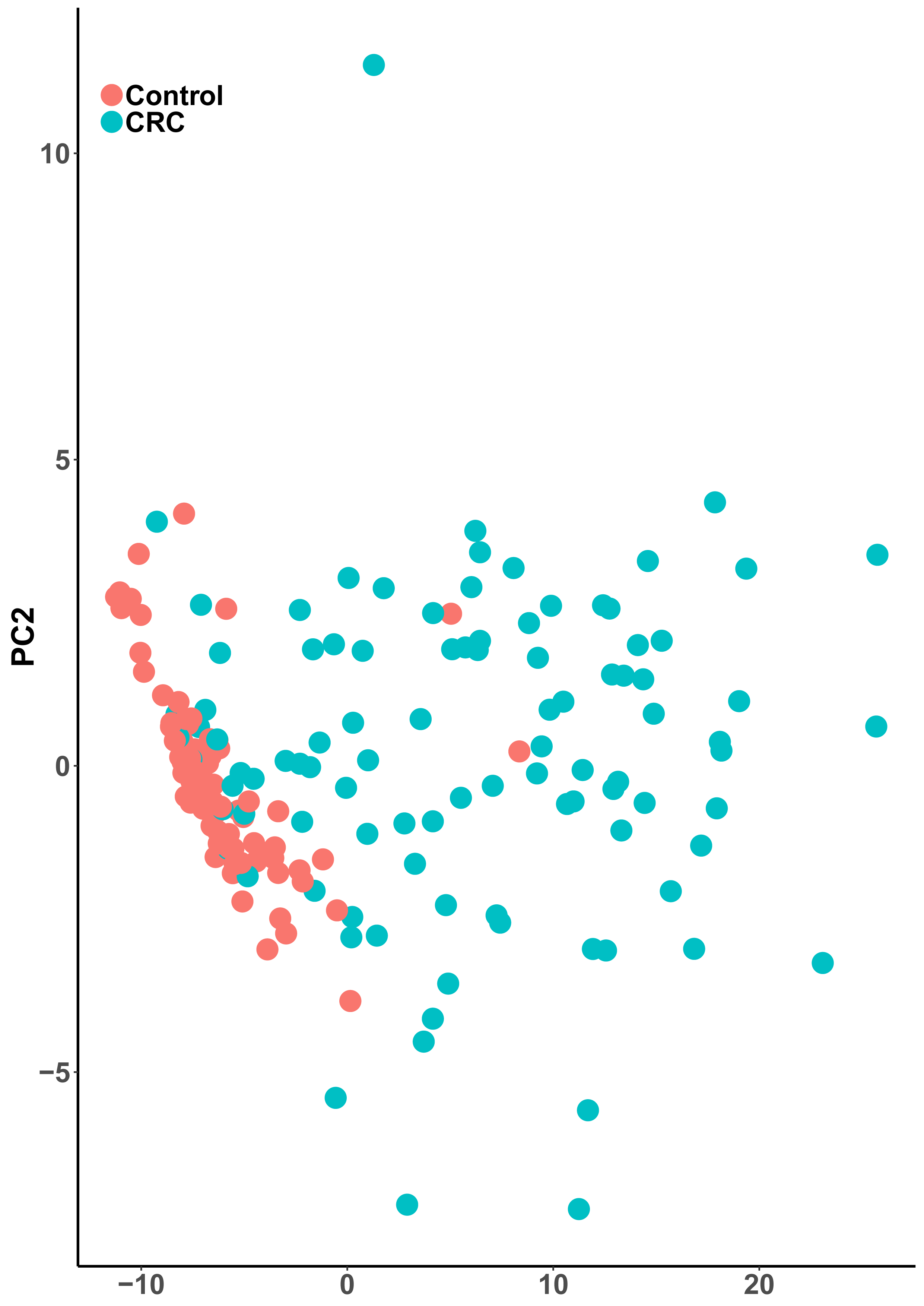

Supplement: Supplementary Figure 3 — Principal Component Analysis of the methylation profiles of the samples in replication cohort 1. All of the CpG sites were preprocessed and simplified into the two-variable space through principal component analysis. The green and red dots represented the CRC tumors and paired controls in replication cohort 1. [file Image_3.JPEG]

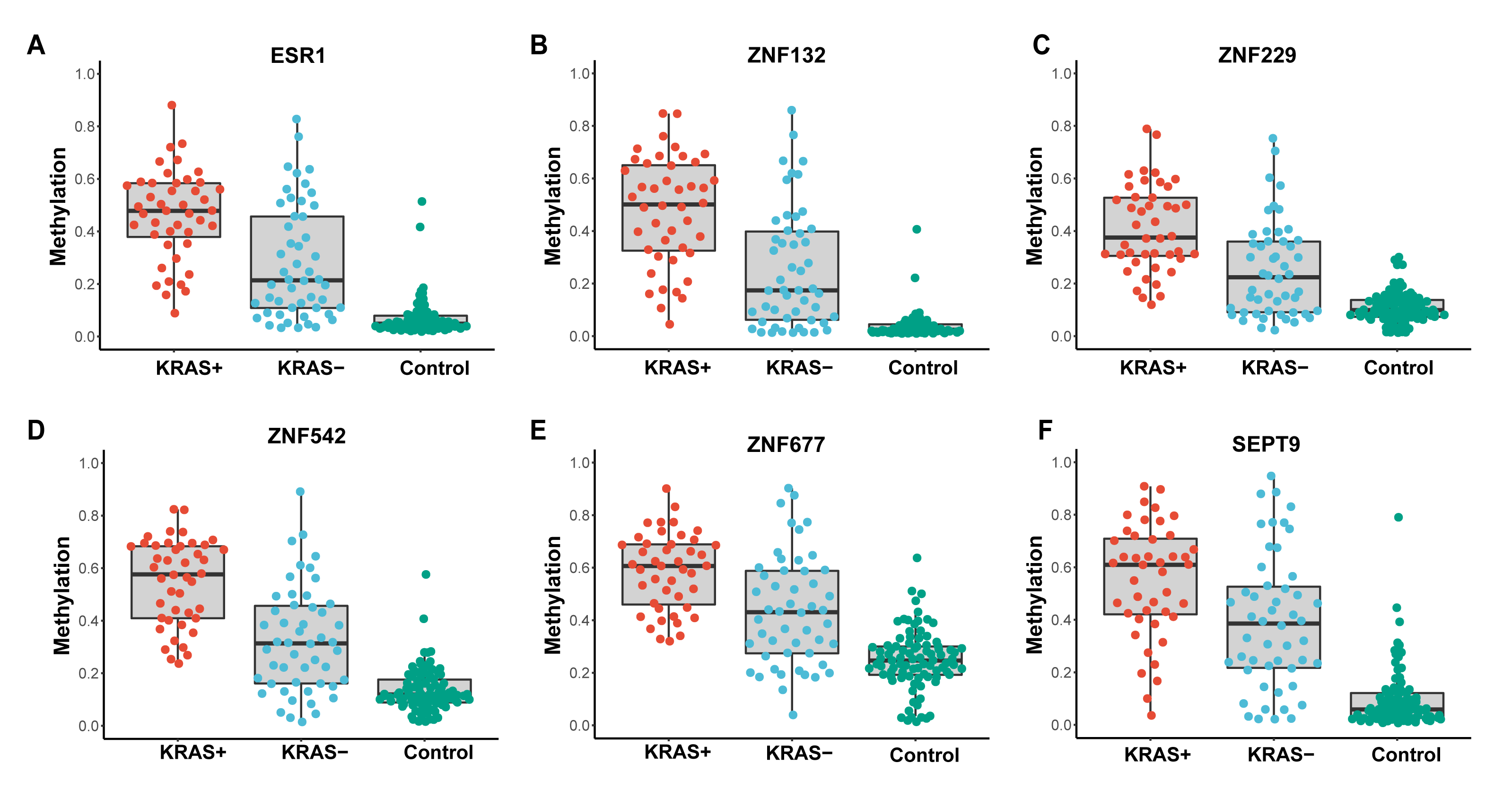

Supplement: Supplementary Figure 4 — Boxplot of the methylation rate of the KRAS +, KRAS- and control samples in replication cohort 1. The mean methylation rate of all the CpG sites in each candidate gene of each sample was depicted as one dot in the boxplot. [file Image_4.JPEG]

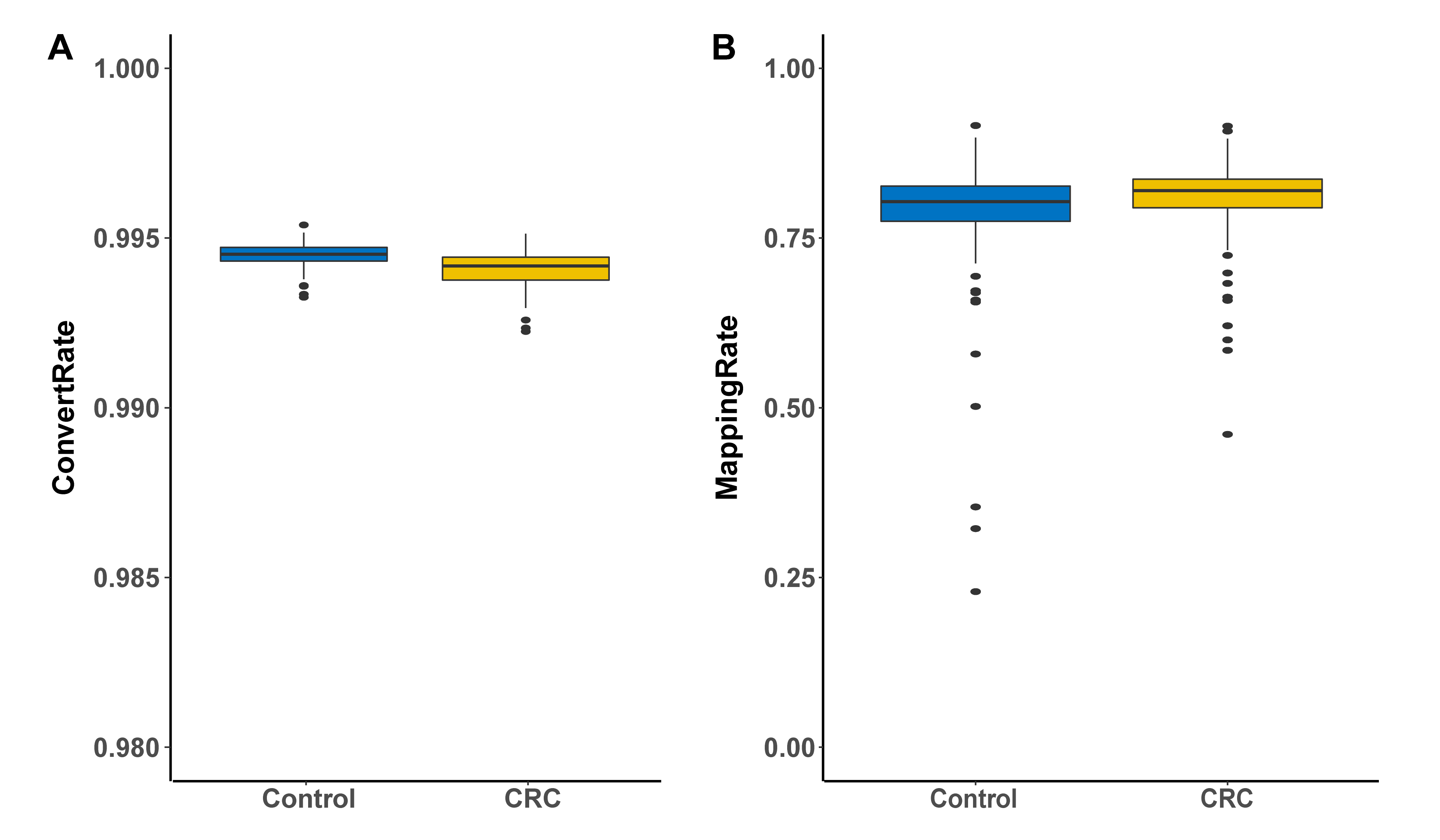

Supplement: Supplementary Figure 5 — Bisulfite conversion rate and mapping rate between CRC tumors and para-tumors. Panels (A,B) represent the bisulfite conversion rate and the reads mapping rate of the samples in replication cohort 2, respectively. [file Image_5.JPEG]

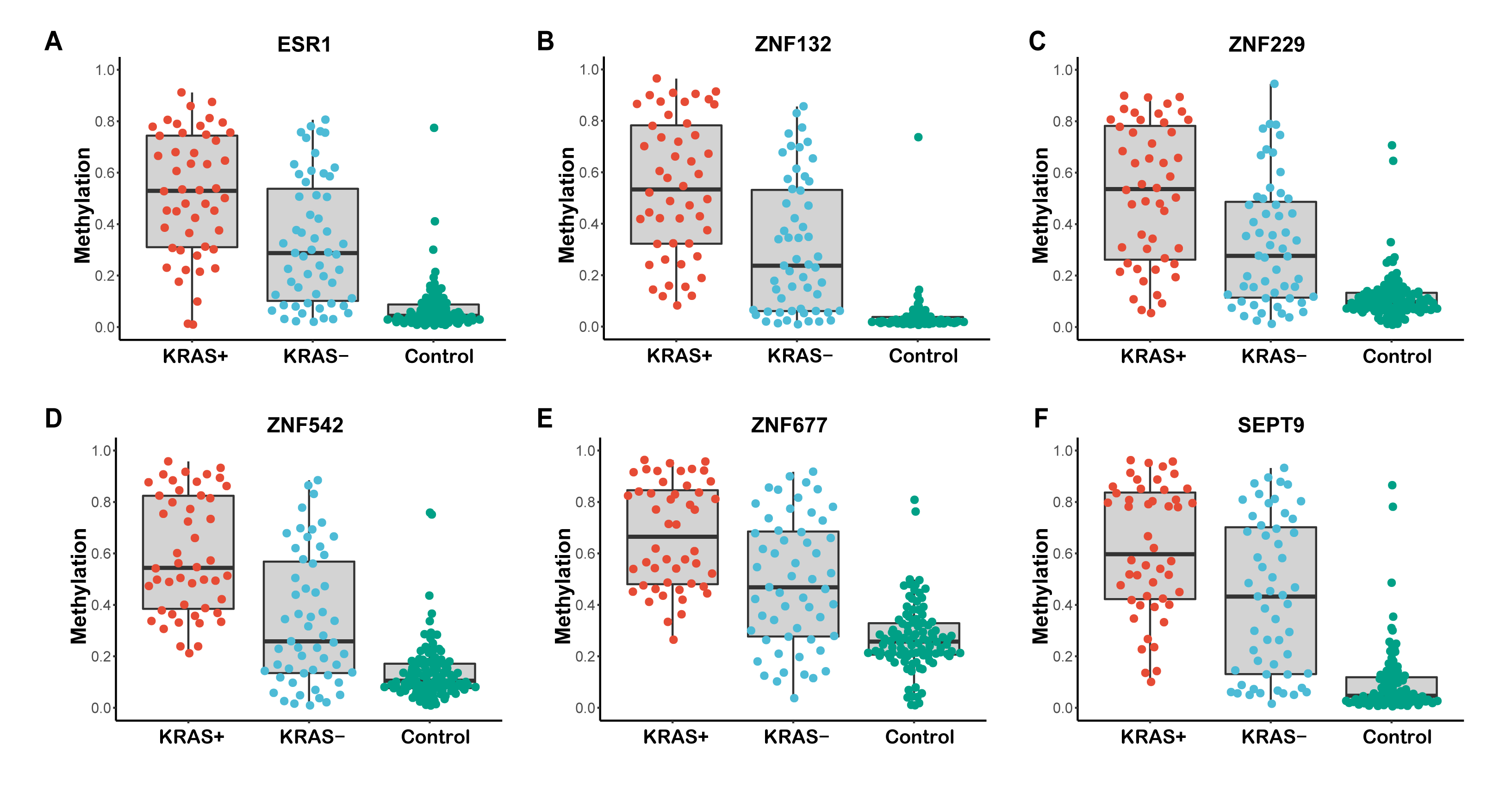

Supplement: Supplementary Figure 7 — Boxplot of the methylation rate of the KRAS +, KRAS- and control samples in replication cohort 2. The mean methylation rate of all the CpG sites in each candidate gene of each sample was depicted as one dot in the boxplot. [file Image_7.JPEG]
